# Endocrine Disrupting Chemicals in Polycystic Ovary Syndrome: The Relevant Role of the Theca and Granulosa Cells in the Pathogenesis of the Ovarian Dysfunction

**DOI:** 10.3390/cells12010174

**Published:** 2022-12-31

**Authors:** Malgorzata Jozkowiak, Hanna Piotrowska-Kempisty, Dominik Kobylarek, Natalia Gorska, Paul Mozdziak, Bartosz Kempisty, Dominik Rachon, Robert Z. Spaczynski

**Affiliations:** 1 Department of Toxicology, Poznan University of Medical Sciences, Dojazd 30, 60-631 Poznan, Poland; 2 Doctoral School, Poznan University of Medical Sciences, Bukowska 70, 60-812 Poznan, Poland; 3 Department of Basic and Preclinical Sciences, Institute of Veterinary Medicine, Nicolaus Copernicus University in Torun, Gagarina 7, 87-100 Torun, Poland; 4 Physiology Graduate Program, North Carolina State University, Raleigh, NC 27695, USA; 5 Prestage Department of Poultry Sciences, North Carolina State University, Raleigh, NC 27695, USA; 6 Division of Anatomy, Department of Human Morphology and Embryology, Wroclaw Medical University, Chalubinskiego 6a, 50-368 Wroclaw, Poland; 7 Department of Veterinary Surgery, Institute of Veterinary Medicine, Nicolaus Copernicus University in Torun, Gagarina 7, 87-100 Torun, Poland; 8 Department of Clinical and Experimental Endocrinology, Medical University of Gdansk, Debinki 7, 80-211 Gdansk, Poland; 9 Center for Gynecology, Obstetrics and Infertility Treatment Pastelova, Pastelowa 8, 60-198 Poznan, Poland

**Keywords:** polycystic ovary syndrome, granulosa cells, theca cells, endocrine disrupting chemicals

## Abstract

Polycystic ovary syndrome (PCOS) is the most common heterogeneous endocrine disorder among women of reproductive age. The pathogenesis of PCOS remains elusive; however, there is evidence suggesting the potential contribution of genetic interactions or predispositions combined with environmental factors. Among these, endocrine disrupting chemicals (EDCs) have been proposed to potentially contribute to the etiology of PCOS. Granulosa and theca cells are known to cooperate to maintain ovarian function, and any disturbance can lead to endocrine disorders, such as PCOS. This article provides a review of the recent knowledge on PCOS pathophysiology, the role of granulosa and theca cells in PCOS pathogenesis, and the evidence linking exposure to EDCs with reproductive disorders such as PCOS.

## 1. Introduction

Polycystic ovary syndrome (PCOS), also known as Stein-Leventhal syndrome, is the most commonly occurring chronic endocrine disorder among women of reproductive age [1]. This condition, with a broad spectrum of heterogeneous syndromes, affects the health of a significant portion of the female population worldwide. PCOS is a complex endocrinopathy encompassing a constellation of various symptoms, such as menstrual abnormalities, infertility, acne, hirsutism, and several metabolic disorders. Considering the varied clinical manifestations, unknown etiology, and complicated pathophysiology, the diagnosis of PCOS still remains a matter of controversy. The prevalence of PCOS is frequently estimated to be 2 to 26% [2,3]. The mentioned divergence in the prevalence rate might result from differences in diagnostic criteria, sample heterogeneity, socioeconomic status, access to medical care, and general health awareness [3]. Furthermore, considering the multiple PCOS phenotypes and the fact that PCOS is being diagnosed mainly by gynecologists and endocrinologists, while it is poorly understood in other specialties and primary care, these estimations may sometimes be understated. It is also known that racial and ethnic differences might be involved in the clinical manifestation of PCOS, due to genetic and environmental susceptibility to endocrinopathies and metabolic diseases. Engmann et al. have revealed that Hispanic women presented a severe PCOS phenotype with more pronounced hyperandrogenism and metabolic abnormalities [4].

Up to now, three sets of standardized diagnostic criteria have been established. In 1990, the National Institutes of Health (NIH) criteria were proposed, defining PCOS as the presence of clinical and/or biochemical hyperandrogenism, oligo/amenorrhea, and anovulation, after the exclusion of related disorders [5]. The most relevant and widely used are the Rotterdam criteria, formulated in 2003. According to them, a clinical diagnosis of PCOS requires the presence of two of the following conditions: (i) oligoovulation or anovulation, (ii) clinical or biochemical hyperandrogenism, (iii) polycystic ovary morphology (PCOM), defined as 12 or more follicles in each ovary measuring 2–9 mm visible on ultrasound [6]. Moreover, two new phenotypes of PCOS were created: (i) ovulatory women with PCOM and clinical and/or biochemical hyperandrogenism, (ii) oligo- and anovulatory women with PCOM without androgen excess and/or hirsutism [6]. According to Zhang et al., based on the new set of criteria, the prevalence of PCOS could increase in the population of women of reproductive age, possibly by as much as 50% [7]. In 2006, the Androgen Excess Society (AES) revised the recent diagnostic criteria and expressed a preference that PCOS should initially be considered as an endocrinopathy with androgen excess or hyperandrogenism [6]. The Rotterdam PCOS diagnostic criteria were supported by the “International evidence-based guideline for the assessment and management of polycystic ovary syndrome” [8]. Based on the revised recommendations, while both oligo- or anovulation and hyperandrogenism are present and related disorders are excluded, an ultrasound is not necessary for diagnosis in adults [8]. The evidence-based recommendations regarding hyperandrogenism have also been highlighted. It is strongly recommended to use calculated free testosterone, free androgen index, or calculated bioavailable testosterone to determine biochemical hyperandrogenism. Furthermore, a comprehensive physical examination should be performed to assess the manifestations of clinical hyperandrogenism, including hirsutism, acne, and alopecia [8]. Hirsutism should be diagnosed using standardized visual scales, such as the modified Ferriman Gallwey (mFG) score [9]. However, ethnicity must be considered to avoid inaccurate clinical assessment, as higher cut-off values have been described in Chinese women, compared to White and Black women [10,11]. 

The diagnostic assessment of PCOS in adolescents may also need a careful approach. The adolescent consensuses support the use of NIH criteria, which include hyperandrogenism and menstrual irregularities/ovulatory dysfunction after exclusion of related conditions [12]. The Rotterdam criteria should not be used, as pelvic ultrasound is not recommended for the diagnosis of PCOS in adolescents. Several studies showed that PCOM commonly occurs in the early years post-menarche in healthy adolescents [8,13,14,15]. Furthermore, international evidence-based guidelines do not recommend using ultrasound for the diagnosis of PCOS in patients with a gynecological age of <8 years [13].

Additionally, the criterion of menstrual irregularity in adolescents was also redefined [13]. Irregular menstrual cycles were defined as normal in the first year after menarche [8]. Furthermore, based on criteria published by the American Academy of Pediatrics and the American College of Obstetrics and Gynecology, the presence of persistent menstrual cycles >45 days during the six years after menarche should be defined as oligomenorrhea [16]. Moreover, primary amenorrhea by the age of 15 or after the three years post thelarche should be considered as a feature of adolescent PCOS within the criterion of irregular menstrual cycles [8].

Recently, an important insight into the Rotterdam criteria has been provided. Carmina and Lobo have indicated the importance of obesity as a characteristic frequently associated with PCOS patients, which currently remains out of the diagnostic criteria for PCOS [17]. The authors have suggested the differentiation of the patients of each Rotterdam PCOS phenotype into two subtypes: obese and lean patients. The modified classification, considering body weight, associated with metabolic alternation or a normal metabolic pattern, may facilitate the process of clinical diagnosis and, in consequence, improve treatment outcomes [17,18].

However, PCOS remains a diagnosis of exclusion. There are several disorders with manifestations similar to PCOS, causing oligoovulation/anovulation and hyperandrogenism that should be excluded. It is essential to rule out disorders such as hyperprolactinemia, nonclassical congenital adrenal hyperplasia, Cushing’s disease, and androgen-secreting tumors [19].

Although the diagnostic criteria of PCOS are widely described, its etiology remains unclear. However, this endocrinopathy can be considered to encompass numerous genetic interactions or predispositions, as well as environmental factors, all contributing to the eventual PCOS phenotype [20,21]. The androgen excess in prenatal/prepubertal life has been suggested to be a reason for the manifestation of PCOS in adulthood [22,23]. Environmental factors play an important role in the development of the epidemic of PCOS in contemporary society, and interest in the possible health concerns posed by EDCs is increasing. According to the Scientific Statement of the Endocrine Society, EDCs play a cardinal role in the etiology of complex metabolic syndromes, such as obesity, diabetes mellitus, and cardiovascular disease [24]. The EDCs were defined by the U.S. Environmental Protection Agency (EPA) as “exogenous agents that interfere with synthesis, secretion, transport, metabolism, binding action, or elimination of natural blood-borne hormones that are present in the body and are responsible for homeostasis, reproduction, and developmental process” [24]. There is accumulative evidence that EDCs are associated with many reproductive disorders among women. To date, these substances have been revealed to impact female and male reproduction, the development of breast and prostate cancers, and play a role in the etiology of complex disorders, such as diabetes, obesity, and cardiovascular disease [24]. Therefore, exposure to environmental EDCs can also contribute to the pathogenesis of PCOS. Due to the extensive occurrence of these compounds in the environment, it is an area of intensive investigation. 

This article provides a review of the recent knowledge on PCOS pathophysiology, the role of granulosa and theca cells in PCOS pathogenesis, and the evidence linking exposure to EDCs with reproductive disorders such as PCOS.

## 2. Pathophysiology of Polycystic Ovary Syndrome

Genome-wide association studies (GWAS) were an important milestone in PCOS genetics. The role of GWAS is to look for associations between common genetic polymorphisms and diseases, providing information about gene loci linked to the trait. GWAS have provided the entire genome search for susceptibility loci for PCOS and its quantitative features [25]. To date, genome-wide association studies have been conducted in Chinese, Korean, and European cohorts and have pointed out the following genetic loci in genes associated with PCOS, e.g., *DENND1A, insulin receptor* (*INSR*), *YAP1*, *C9orf3*, *RAB5B*, *HMGA2*, *TOX3*, *SUMO1P1/ZNF217*, *THADA*, *FSHR*, and *LHCGR* [26,27]. Moreover, mentioned genome research has provided insights into several biological pathways essential for PCOS pathogenesis, which can be disrupted, involved in androgen and gonadotropin secretion, and cell survival [28,29,30].

Furthermore, based on biochemical and genotype data from a previously performed GWAS [30], Dapas et al. have investigated the phenotypic subtypes of PCOS using an unsupervised hierarchical cluster analysis in a genotyped cohort of 893 PCOS women and then replicated the clusters in a cohort of 263 independent, ungenotyped PCOS cases [31]. Interestingly, the research has indicated two PCOS subtypes: (i) a reproductive group (21–23%), characterized by higher levels of LH and SHBG as well as relatively low BMI and insulin levels; (ii) a metabolic group (37–39%), described by higher BMI, glucose, and insulin levels, accompanied by lower LH and SHBG levels [31]. Moreover, a significantly higher number of PCOS patients from the reproductive subtype were found to carry at least one of the previously described deleterious *DENND1A* variants, as compared to women with other PCOS subtypes [31].

Overall, these results demonstrate that the reproductive and metabolic subtypes appear to have a distinct genetic architecture and are associated with different underlying biological mechanisms. It might be of high significance since the patients from the mentioned subtypes may respond differently to therapy.

### 2.1. Neuroendocrine Dysfunctions and Reproductive Abnormalities in PCOS

Physiological ovarian follicular development consists of various subtle mechanisms as well as metabolic and intraovarian interactions, which all contribute to the ovulation of one dominant antral follicle during the menstrual cycle. Hence, in PCOS, follicle growth is frequently disrupted by hyperandrogenism, insulin resistance, leading to hyperinsulinemia, and dysfunction of intraovarian paracrine signaling [32]. The accumulation of prematurely arrested small antral follicles within the ovarian cortex and subsequent failure of dominant follicle development result in PCOM. The mentioned follicular arrest in PCOS is clinically manifested by menstrual irregularity and anovulation [32].

Gonadotropin abnormalities are one of the major issues in PCOS pathophysiology. Overall, 70% of women with PCOS are estimated to manifest increased serum immune and bioactive LH levels [23].

Moreover, the hyperresponsiveness of the theca cells to LH stimulation leads to enhanced ovarian androgen production and, consequently, hypothalamic–pituitary axis dysfunction. In addition, the compensatory aromatization to estrogens in granulosa cells (GCs) is diminished due to the reduced FSH levels. Higher LH pulse amplitude and frequency contribute to the significantly elevated LH:FSH ratio. It is the result of increased hypothalamic gonadotropin-releasing hormone (GnRH) pulsatile release, associated with a reduced steroid hormone negative feedback loop of LH secretion due to androgen excess [33,34,35]. Furthermore, hyperandrogenism has been reported to reduce the sensitivity of gonadotropic hypothalamic cells to estradiol and progesterone, enhancing GnRH and LH secretion [36]. Thus, treatment with an androgen receptor blocker, such as flutamide, has been shown to improve the sensitivity of the GnRH pulse generator among women suffering from PCOS within four weeks [37]. Subsequent studies have revealed that androgen excess elevates initial recruitment of the primordial follicles, initiates premature luteinization, and inhibits selection of the dominant follicle [38,39,40,41]. Hence, hyperandrogenism is suggested to be the underlying cause of PCOM. Moreover, genetic factors have also been proposed to potentially contribute to PCOM due to ovarian tissue predisposition for hypersecretion of androgens, as a result of mutations in the androgen receiver, sex hormone-binding globulin (SHBG), and steroidogenic enzyme genes [42].

According to the available literature and in vivo studies, it has been suggested that functional ovarian hyperandrogenism (FOH) is the crucial derangement in PCOS. Mostly otherwise unexplained steroidogenic hyperactivity appears to be a fundamental disruption of intraovarian processes and, subsequently, ovarian androgen and estrogen secretion [43]. Androgen excess is frequently considered to be essential in PCOS, since circulating total and free testosterone and dehydroepiandrosterone sulfate (DHEAS) levels are significantly elevated in the majority of PCOS cases [44]. The clinical manifestations of hyperandrogenism are hirsutism, acne, and alopecia. However, 15–20% of women with clinical hyperandrogenism were not diagnosed with this endocrinopathy [45,46]. Over 60% of women suffering from PCOS have functionally typical FOH, described by 17-hydroxyprogesterone hyperresponsiveness to stimulation with gonadotropin. The remaining PCOS cases are characterized by FOH, which presents with an elevated testosterone level after suppression of adrenal androgen production or isolated functional adrenal hyperandrogenism [47].

### 2.2. Metabolic Disorders in PCOS

It is well known that PCOS is a multifactorial metabolic syndrome. The most critical metabolic feature in the clinical manifestation of PCOS is peripheral insulin resistance, resulting in compensatory hyperinsulinemia. Insulin resistance is estimated to affect 60–80% of women diagnosed with PCOS, and occurs independently of obesity [48]. Insulin resistance could be described as the failure response to regular circulating levels of insulin, which contributes to the pathogenesis of T2DM, hypertension, atherosclerosis, hyperlipidemia, and other metabolic syndromes. It has been suggested that compensatory hyperinsulinemia, as a response to insulin resistance, leads to hyperandrogenism through stimulation of ovarian androgen secretion and an inhibitory effect on liver SHBG production [32,49,50]. Although multiple molecular explanations for the underlying insulin resistance in PCOS have been proposed, the main mechanism remains elusive. There is a large body of evidence suggesting primary derangements in the insulin-mediated glucose transport, abnormal GLUT4 expression, and insulin or adrenergic-regulated lipolysis in adipose tissue, regardless of normal insulin binding [49,51,52,53,54]. Therefore, insulin resistance is known to be associated with adiposity, as evidenced by the higher prevalence of obesity among women suffering from PCOS than healthy women of the same age group [55]. The mechanism of PCOS-related insulin resistance contributes to fundamental, tissue-specific derangements in intracellular signaling by paracrine, autocrine, and endocrine factors, affecting particular metabolic pathways [47]. At the molecular level, abnormalities in the phosphorylation of the insulin receptor, or insulin-receptor substrate, have been suggested to be the most prominent among the mechanisms of insulin resistance in PCOS. Intracellular serine kinases phosphorylate the insulin receptor and insulin receptor substrate-1, leading to reduced activation of the phosphatidylinositol-3-kinase signaling pathway, and therefore inhibiting glucose transport. Moreover, serine phosphorylation is also known to activate mitogenic pathways mediated by ERK/MAPK [56]. Interestingly, there are several similarities in the PCOS-related insulin signaling pathway in the ovaries and the other tissues. It has been revealed that phosphorylation of microsomal cytochrome P450c17 by serine kinases leads to increased 17,20-lyase activity. Cytochrome P450c17 is known to catalyze steroid 17-α-hydroxylase activity and scission of the C17-C20 steroid bond (17,20-lyase) at the same active site. Hormonally regulated serine phosphorylation of cytochrome P450c17 has been proposed to be an etiologic connection between hyperandrogenism and insulin resistance in PCOS [57]. In addition, recent studies have indicated irregular phosphorylation of glycogen synthase kinase 3 and serine/threonine-protein kinase AKT in fibroblasts, adipocytes, and myocytes in women suffering from PCOS [49,52,58,59]. The derangements in insulin secretion and activity significantly increase the risk for the development of various metabolic disorders. Consequently, 30–40% of women with PCOS struggle with impaired glucose tolerance, 30–70% suffer from obesity, and 10% have type 2 diabetes by the fourth decade of life [56].

Metabolic derangements might also notably increase the risk of cardiovascular dysfunction in PCOS patients. Several studies have revealed that a higher risk of cardiovascular disease is correlated with the severity of PCOS phenotypes in obese and non-obese patients [60,61].

### 2.3. Environmental Factors and PCOS Development

Environmental factors are known to have an important role in PCOS pathogenesis. The accumulating data indicate that socioeconomic status (SES), as well as unhealthy behaviors, such as bad eating habits, smoking, and insufficient physical activity, impact the development of PCOS [62,63]. Studies have indicated the relationship between low SES in childhood and PCOS development in later years [64]. Low SES has also been linked to the prevalence of obesity, one of the major PCOS-associated metabolic comorbidities [65,66]. Therefore, lifestyle can affect PCOS phenotypic expression. In fact, the risk of PCOS development seems to be greater in obese women. It has been revealed that weight gain exacerbates the metabolic and reproductive dysfunctions of PCOS, which are manifested by worsened insulin resistance, abdominal obesity, irregular menstrual cycles, and hyperandrogenism in the most severe PCOS phenotype [67,68,69,70,71]. For instance, Carmina et al. suggested that the widely described, more pronounced metabolic dysfunctions in PCOS women in the USA, compared to PCOS women in other countries, might be relatively connected with higher body weight and dietary saturated fat intake [72]. On the other hand, reducing body weight contributes to lower circulating androgen and insulin levels, ameliorates ovulatory and menstrual aberrations, and improves dyslipidemia [73,74,75,76,77]. Similarly, regular moderate-intensity exercise training decreases body adipose tissue and improves insulin sensitivity even without calorie restriction and weight loss [78].

Exposure to EDCs is certainly an important predisposing environmental factor contributing to PCOS development [79]. Therefore, EDCs have been reported to target metabolic and reproductive function, leading to abnormalities that resemble those of PCOS. Bisphenol A (BPA) is one of these compounds primarily used in the production of polycarbonate plastics. BPA is found in numerous products, including water bottles, medical devices, and dental sealants [80,81]. Animal studies have suggested that BPA disrupts the hormonal balance due to enhancing androgen production in vitro [82], and inducing insulin resistance in vivo [83]. Several studies have reported that BPA accumulates in women with PCOS at elevated levels, leading to androgen excess, which in turn decreases its hepatic clearance [84,85].

Growing interest has been devoted to the impact of EDCs on the altered composition of gut microbiota (GM), known as dysbiosis. GM is a complex population of microorganisms (bacteria, archaea, and eukarya) that colonize the human gastrointestinal tract, fulfill many critical roles in essential host functions, and therefore influence human health and diseases [86]. The main taxonomic phyla residing in the intestine include Firmicutes, Bacteroides, Proteobacteria, Fusobacteria, and Verrucomicrobia [87]. Many recent articles and reviews have highlighted the various physiological functions of GM, including maintenance of intestinal mucosal barrier integrity, regulation of host immunity, modulation of immune development, protection against pathogens, and synthesis of essential vitamins that the host is incapable of producing [86]. Due to its large genomic content (microbiome), GM offers many benefits to the host, and the diversity of the microbial population is of great importance since it might be considered a functional expansion of host genomes [88].

Recently, several clinical and experimental studies showed that exposure to EDCs significantly altered the composition of gastrointestinal bacteria [86]. It has therefore been revealed that GM is involved in xenobiotic biotransformation and has the capacity to extensively metabolize EDCs, which might change or modulate their toxicity for the host [89,90]. Current research suggests a significant correlation between GM composition and female reproductive health [86]. GM has been suggested to influence female fertility by altering the level of sex hormones [91]. Furthermore, reduced GM biodiversity in both the gut and reproductive tract may lead to immune abnormalities, impaired immunosurveillance, and affected immune cell profiles. Dysbiotic GM has been observed in various infertility-related disorders such as endometriosis, PCOS, insulin resistance, and obesity [92,93,94,95,96,97]. For instance, an abnormal Escherichia:Shigella ratio and an excess of Bacteroides have been revealed in PCOS patients compared to healthy women [95].

There are numerous sources of daily human exposure to EDCs, including food and beverages, air, dust, and water [98]. Therefore, there is growing concern about the negative impact, possibly caused by these compounds, on women’s reproductive health, since exposure to these chemicals might exaggerate the severity of the PCOS phenotype.

## 3. The Role of Granulosa and Theca Cells in PCOS

### 3.1. Granulosa and Theca Cells—Two Cell, Two Gonadotropin Theory

GCs are widely considered a critical somatic part of the ovary. GCs surround the oocyte, promote oocyte development, produce sex steroids and growth factors, and overall contribute to normal folliculogenesis and menstrual cycle [99]. GCs can be divided into two types, mural GCs and cumulus cells, which transform from each other at pre-antral to antral follicle transition. Mural GCs consist of the external layer of lining the follicle, whereas cumulus cells adhere to the developing oocyte. Further, GCs aromatize androgens, produced by neighboring theca cells, during folliculogenesis [100]. Theca cells are endocrine cells that differentiate from the interfollicular stroma in response to factors secreted by the growing follicles. Any disturbance in the complex processes in GCs and theca cells may lead to endocrine disorders, such as PCOS, or even cause infertility. 

**Figure 1:**
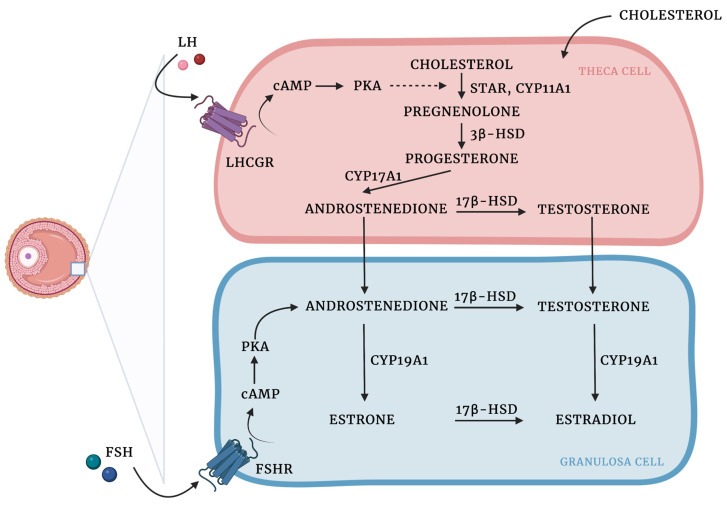
Ovarian steroidogenesis: two cell, two-gonadotropin theory. Ovarian steroids are synthesized from cholesterol, which diffuses from the circulation into theca cells and is mobilized into mitochondria by steroidogenic acute regulatory protein (STAR) activity [102]. LH binds to LHCGR on the cell surface, which results in the increased expression of steroidogenic enzymes involved in androgen production. Cholesterol is then converted into pregnenolone by the cholesterol sidechain cleavage enzyme (CYP11A1). In the smooth endoplasmic reticulum, pregnenolone is transformed into progesterone due to the activity of 3β-hydroxysteroid dehydrogenase (3β-HSD). Then, due to the activity of CYP17A1 progesterone is converted to androstenedione, which in turn might be transformed into testosterone by 17β-hydroxysteroid dehydrogenase (17β-HSD) or translocated into the GCs, where aromatase (CYP450arom; CYP19A1) converts androstenedione to estrone and testosterone to estradiol. 17β-HSD might also produce estradiol using estrone as a substrate [103,104,105,106]. Created with BioRender.com.

Granulosa and theca cells are known to cooperate in the biosynthesis of ovarian hormones (Figure 1). This cooperation is described by the two-cell, two-gonadotropin theory, which claims that ovarian steroids are synthesized from cholesterol through complex interactions between the granulosa and theca cells [101].

There is an ongoing discussion on how various EDCs can alter the complexity of the synthesis and metabolism of ovarian steroid hormones [107]. Thus, disruption of the endocrine system occurs when the hormones do not bind to the receptors, and the way hormones elicit their function is changed.

### 3.2. The Role of AMH-Mediated SMAD Signaling Pathway in PCOS

Anti-Müllerian hormone (AMH), a glycoprotein hormone from the TGF-β superfamily, is produced by GCs with the highest expression in the preantral and small antral follicles, and has an important role in folliculogenesis. During the ovary cycle in physiological ovaries, AMH continues to be expressed in growing follicles, playing a crucial role in the arrest of antral follicle development, reducing follicle sensitivity to FSH, and inhibiting recruitment of follicles from the resting pool. When the follicles reach the size at which they are dominant, the production of AMH is timely reduced. AMH is known to be used as a molecular biomarker for the determination of ovarian reserve, but also ovarian dysfunction, such as PCOS [108].

Elevated levels of AMH blood concentration in women with PCOS were recently confirmed by several studies [109,110,111]. Anomalies in follicle growth, resulting in an increased number of small antral follicles, contribute to anovulatory infertility in PCOS women. It has been revealed that serum AMH levels are two to five times higher in PCOS women, and relatively elevated in women presenting anovulatory cycles compared to the ovulatory PCOS phenotype [112,113,114]. Therefore, there is increasing evidence that this derangement in ovarian physiology is associated with unsatisfactory pregnancy outcomes.

Multiple molecular mechanisms have been proposed to explain the impact of AMH on human ovarian GCs. AMH has been shown to reduce follicle responsiveness to FSH due to the downregulation of FSH receptor expression in vitro in human GCs [115] and the expression of aromatase [116]. Interestingly, gonadotropins are also involved in the regulation of AMH expression; FSH has been indicated as a suppressor, and LH has been shown to stimulate AMH expression in the GCs of PCOS women [117,118]. Furthermore, Pierre et al. have revealed that the mRNA expression of *AMH receptor II* (*AMHRII*) is downregulated by LH in GCs from women with regular ovaries, but not those suffering from PCOS [119].

In GCs derived from polycystic ovaries, hyperandrogenism inhibits AMH down-expression through elevated 5α-dihydrotestosterone (5α-DHT) levels, or indirectly through the conversion of testosterone to estradiol and increased expression of ERα [120]. The studies of Dilaver et al. have pointed out for the first time differences in the AMH/AMHRII signaling, associated with the intracellular SMAD signaling pathway, in regular and polycystic ovaries. Prolonged exposure of GCs derived from polycystic ovaries to high levels of AMH has been revealed to affect the expression patterns of aromatase and FSHR and disrupt SMAD signaling by increasing the level of I-SMAD-6, -7, and diminishing activation of SMAD-1/5/8 and co-SMAD-4 [120].

**Figure 2:**
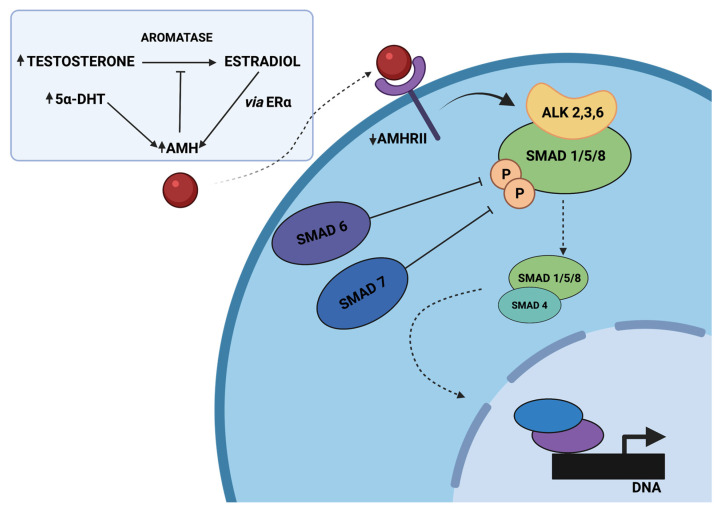
The proposed model of disrupted AMH signaling in women with PCOS, adapted from Dilaver et al. [120]. Hyperandrogenism inhibits the decrease in AMH levels directly by elevated 5α-dihydrotestosterone (5α-DHT) levels or indirectly through the conversion of testosterone to estradiol and the increased expression of ERα. Elevated AMH levels might diminish the expression of aromatase and increase the protein levels of the inhibitory SMADs (SMAD-6, SMAD-7), associated with negative regulation of intracellular SMAD signaling. It might disrupt pSMAD-1/5/8 binding to SMAD-4 and, as a consequence, alter the expression of various genes. Created with BioRender.com.

AMH-mediated SMAD signaling is a complex downstream of events, beginning with AMH binding to the AMHRII transmembrane serine/threonine kinase receptor and activating the Type 1 receptor, which contributes to the phosphorylation of SMAD-1/5/8 proteins. Then, a tetrameric complex of two AMHRII and two Type I receptors (probably ALK 2,3 or 6) is formed, and SMADs-1/5/8 are joined to the common SMAD-4 (co-SMAD-4). The mentioned complexes are translocated to the nucleus, where they alter various genes’ expression due to transcriptional factors, coactivators, and corepressors [121]. In PCOS, the cascade contributing to SMAD signaling is disrupted by high AMH concentration, leading to increased protein levels of the inhibitory SMADs (I-SMAD), associated with negative regulation of intracellular SMAD signaling (Figure 2). SMAD-6 has been revealed to inhibit activation of bone morphogenetic protein (BMP) pathways, altering pSMAD-1/5/8 binding to co-SMAD-4 in the mechanism of competitive inhibition. Furthermore, SMAD-7 is known to inhibit BMP signaling by binding to the type I receptor [122]. Moreover, follicle growth may also be disrupted by reduced expression of AMHRII [120]. 

### 3.3. The Role of the PI3K/AKT/FOXO Signaling Pathway in PCOS

Subsequent studies have confirmed that insulin resistance and impaired glucose metabolism in PCOS are related to the promotion of ovarian GCs apoptosis and follicular development dysfunctions [123,124]. The mechanism of this pro-apoptotic activity is not yet fully understood; however, the role of SH2B adaptor protein 3 (LNK), an important regulator of the insulin signaling pathway, has been suggested. 

LNK is a member of the Src homology 2B (SH2B) family of intracellular adaptor proteins and is known to play an important role in the insulin signaling pathway in the ovary, glucose homeostasis, and reproduction [125]. Furthermore, several studies have also indicated the participation of LNK in the pathogenesis of type 1 diabetes, hypertension, and cardiovascular disease, but also in malignant tumors [126,127,128,129]. In patients with insulin resistance, LNK levels have been revealed to be significantly increased as compared to the control group [130]. The authors suggested that LNK negatively regulates the insulin-activated PI3K/AKT/FOXO3 signaling pathway in GCs and, consequently, promotes GCs derangements and apoptosis, leading to ovulation disorders in PCOS [131]. Phosphatidylinositol 3-kinase (PI3K) signaling is one of the main pathways involved in the regulation of cell proliferation, survival, migration, and metabolism in physiological and pathological processes. Subsequent studies in humans and mice have confirmed that PI3K/AKT signaling and the downstream pro-apoptotic genes (e.g., *FOXO1*, *Bax*, *caspase-9*, *caspase-3*) participate in the regulation of GC growth and apoptosis during follicular development [132,133]. FOXO transcription factors are members of the Fork-head family of proteins and the main direct substrates of the protein kinase AKT following insulin or growth factors stimulation [134]. Among the FOXO subgroup, four members (FOXO1, FOXO3, FOXO4, FOXO6) have been identified in humans [134]. The FOXO family is known to be a key downstream target of PI3K/AKT. 

Normally, insulin binds to the receptor, leading to activation of the PI3K/AKT/FOXO3 signaling pathway, promotes FOXO3 export from the nucleus to the cytoplasm, contributes to inhibition of the expression of pro-apoptotic genes, increasing cell survival, growth, and proliferation [131,135]. Increased LNK levels alter insulin-mediated phosphorylation of AKT and FOXO3, promoting nuclear localization of FOXO3, and consequently leading to enhanced apoptosis in GCs [131]. In vitro studies have also revealed that LNK knockout moderately restores the estrous cycle and improves glucose metabolism in the PCOS mouse model, compared to wild-type PCOS mice [131].

To date, several studies have confirmed derangements in the PI3K/AKT signaling pathway in PCOS patients and animal models of PCOS [136,137]. Gong et al. have suggested that derangements in PI3K/AKT signaling alter the balance between pro- and anti-apoptotic events in GCs. The increased expression of pro-apoptotic *FOXO1*, *Bax, caspase-9*, *caspase-3*, and decreased levels of *PI3K*, *AKT*, and *Bcl-2* have been observed [138]. Moreover, the intracellular ROS level in PCOS GCs was three times higher compared to the control. Interestingly, the study has revealed that growth hormone (GH) significantly decreased ROS production by more than 50%, and decreased the apoptotic rate in PCOS GCs, probably through the activation of PI3K/AKT signaling [138]. In contrast to these findings, several studies have shown enhanced activity of the PI3K/AKT signaling pathway in some PCOS patients [139,140], which might be associated with ethnic differences. Therefore, considering the conflicting results, further research is needed.

### 3.4. The Role of the HMGA2/ IGF2BP2 Signaling Pathway in PCOS

The HMGA2/IGF2BP2 signaling pathway has been indicated to play a critical role in cell proliferation and differentiation [141,142]. *HMGA2* belongs to a family of *HMGA* genes that consist of three DNA-binding domains and an acidic C-terminal tail [143]. An increase in *HMGA2* expression has been observed not only during embryonic development but also in various cancers, suggesting its role in controlling cell proliferation [144]. Insulin-like Growth Factor 2 mRNA Binding Protein (IGF2BP2) plays a vital role in metabolism, and the variants in this gene have been associated with susceptibility to T2DM [145].

Recent studies have revealed that mRNA levels of *HMGA2*, a proposed GWAS susceptibility locus, and *IGF2BP2* expression were significantly increased in GCs derived from women with PCOS compared with controls [146]. In KGN and SVOG cell lines, the HMGA2/IGF2BP2 signaling pathway has been shown to regulate the expression of the *CCND2* and *SERBP1* genes, which are involved in promoting cell proliferation. Interestingly, the mRNA, as well as protein levels of CCND2 and SERBP1 were also elevated in the GCs of PCOS women, leading to enhanced proliferation and decreased apoptosis. Taken together, the studies suggest that overexpression of HMGA2 and increased activity of the HMGA2/IGF2BP2 signaling pathway in ovarian GCs promote cell proliferation and, consequently, the PCOM [146]. 

### 3.5. The Role of Theca Cells in PCOS Development

Studies conducted in the past decade have built a convincing argument that ovarian theca cells are the main source of excess androgen secretion in women suffering from PCOS [147,148,149]. Therefore, it has been revealed that thecal tissue or theca cell cultures derived from women with PCOS secrete significantly higher amounts of androgens compared to cultures derived from healthy women [148,150,151].

In vitro studies have revealed that derangements in theca cell functions are associated with androgen excess and abnormal steroid secretion in response to gonadotropin stimulation [152]. It has been shown that progesterone, 17-hydroxyprogesterone, and testosterone secretion were significantly increased in theca cell cultures derived from PCOS patients [151,152]. Furthermore, studies have revealed a remarkably enhanced metabolism of precursors (basal and cyclic AMP-stimulated pregnenolone, progesterone, and dehydroepiandrosterone) into testosterone, associated with increased androgenic 17β-HSD activity. Moreover, increased mRNA expression of *CYP11A*, *CYP17A1*, *P450c17*, *3β-HSD*, and 17β-HSD enzyme activities were noted in PCOS theca cells compared to normal cells [152]. *CYP17A1* and *CYP11A1* genes encode the pivotal enzymes associated with androgen biosynthesis in theca cells: steroid-17-α-hydroxylase/17,20 lyase and cholesterol side-chain cleavage enzyme, respectively [151,153,154,155]. Thus, increased expression of the mentioned enzymes in women with PCOS enhances androgen biosynthesis by theca cells. Recently, increased activity of P450c17 and 3β-HSD has also been revealed to play a crucial role in the increased synthesis of testosterone precursors, and consequently increased androgen secretion in PCOS by theca cells [153].

*DENND1A* is a member of the family of 18 human genes called “connecdenns” and encodes a protein that has been identified as a guanine nucleotide exchange factor converting inactive GDP-bound Rab35 into its active GTP-bound form. Genetic alterations within the *DENND1A* gene have been noted in PCOS. Furthermore, the *DENND1A* locus at 9q22.32 has been identified in both Asian and European populations [156,157,158,159]. Thus, *DENND1A* might be considered a strong PCOS susceptibility gene [160]. McAlisster et al. have revealed that *DENND1A.V2*, a splice variant derived from the *DENND1A* gene, plays a pivotal role in theca cell steroidogenesis. Overexpression of *DENND1A.V2* results in the expression of the *CYP17A1* and *CYP11A1* genes and, consequently, increased androgen secretion. Moreover, recent studies have indicated that knock-down of the *DENND1A.V2* gene in PCOS theca cells diminished androgen secretion due to decreased *CYP17A1* and *CYP11A1* genes transcription, restoring the normal phenotype of theca cells, which confirmed the role of *DENND1A* in hyperandrogenism associated with PCOS [152].

However, the mechanism of DENND1A.V2 steroidogenic activity is not fully understood. Since DENND1A is one of the proteins involved in protein trafficking, clathrin-mediated endocytosis, and receptor recycling, it might be suggested that DENND1A alters LH action due to LH receptor signaling upregulation [28,47].

Moreover, according to the genotype-phenotype assessment performed by Tian et al., PCOS susceptibility variants in the *THADA* and *INSR* genes are associated with a higher risk of metabolic syndrome in women suffering from PCOS, while variants in *DENND1A* and *TOX3* increase the risk of insulin resistance [161].

### 3.6. The Role of Circadian Rhythm in PCOS Development

In recent years, several studies have confirmed that light exposure and sleep disturbance are associated with acute circadian misalignment, which consequently contributes to the development of metabolic diseases and fertility impairment [162,163]. Interestingly, it has been suggested that circadian rhythm, which orchestrates the physiological functions of the body, could be one of the contributing factors to androgen excess in patients with PCOS [163]. Therefore, Wang et al. have observed a significant association between long-term night shift work and PCOS [163]. 

Recently, Johnson et al. have suggested that circadian rhythm is one of the factors contributing to androgen excess in PCOS due to its role in altering peripheral androgen metabolism [164]. In fact, the study demonstrated increased mRNA levels of steroidogenic enzymes: STAR, CYP17A1, and aldo-keto reductase family 1 member C3 (AKR1C3). The *AKR1C3* is known to encode 17β-hydroxysteroid dehydrogenase type 5 that converts androstenedione to testosterone. Furthermore, different expression patterns of *steroid 5-alpha-reductase 1 and 2* (*SRD5A1* and *SRD5A2*) were observed in patients with PCOS [164]. The *androgen receptor* (*AR*) transcript level was also elevated in the peripheral blood mononuclear cells (PBMCs) of women with PCOS. In contrast, the authors found a decrease in CYP19A1, a key factor responsible for estrogen synthesis, in women with PCOS compared to healthy women [164]. 

Interestingly, the expression of the steroidogenesis genes was shown to vary between PCOS phenotypes. The most significant differences in transcript levels were observed in phenotype A (hyperandrogenism, ovulatory dysfunction, polycystic ovaries), while in phenotype D (ovulatory dysfunction, polycystic ovaries), the changes were less pronounced. It might be a result of heterogeneity as well as a different presentation of the clinical and biochemical characteristics of PCOS cases [164].

Circadian rhythm is known to be modulated through several transcriptional and post-translational autoregulatory feedback loops. The study has shown downregulation of transcript levels of circadian locomotor output cycles kaput (*CLOCK*), brain and muscle aryl hydrocarbon receptor nuclear translocator-like 1 (*BMAL1*), and neuronal PAS domain protein 2 (*NPAS2*) in PBMCs, as well as significantly decreased CLOCK protein expression in women with PCOS [164]. The mRNA expression profiles of the circadian genes *BMAL1* and *PER1* were also altered after darkness treatment in rats [165]. 

Heterodimers of CLOCK, BMAL1, and NPAS2 act as transcriptional factors that activate the promoter sequences of the repressor genes-cryptochrome circadian regulators (CRY1 and CRY2) and period circadian regulators (PER1, PER2, and PER3). Once the PER/CRY heterodimer reaches a critical level, the proteins are translocated to the nucleus where CRYs repress CLOCK-BMAL1-induced transcription. CRYs and PER are therefore negative regulators, while CLOCK-BMAL1 is the positive arm of the feedback loop [164]. In the GCs of PCOS patients, it has been shown that there is decreased expression of *BMAL1*, which contributes to aromatase expression, and consequently there is reduced estrogen synthesis [166]. The study of Johnson et al. has revealed increased expression of mRNA levels of negative regulators of circadian pathway genes *(PER1, PER2, CRY1, CRY2*, as well as *DEC1* and *DEC2*) in the PCOS group compared to controls [164]. 

Retinoic acid receptor-related orphan receptor α (RORα) and the nuclear orphan receptor α (REV-ERBα) are other key regulators of BMAL1, the secondary feedback loop in the circadian cycle [167]. On the one hand, the transcription of REV-ERBα is activated by the BMAL1/CLOCK heterodimer; on the other hand, it is repressed by CRY/PER which results in circadian oscillations of REV-ERBα. Moreover, REV-ERBα and REV-ERBβ are known to repress the transcription of BMAL1/CLOCK and BMAL1, respectively [168]. 

The study of Sun et al. has shown that the expression of REV-ERBα and REV-ERBβ is significantly downregulated in the GCs derived from PCOS patients compared to healthy women [169]. REV-ERBs have been revealed to play an important role in various metabolic, neuronal, and inflammatory processes, as well as in lipid homeostasis [169]. Genetic knock-out experiments have, in turn, explained the meaning of these proteins in the circadian cycle; the expression of *BMAL1* and *CLOCK* in Rev-erbα-deficient mice was significantly increased when compared with wild-type mice [170], and Rorα– and Rorβ-deficient mice were found to display an abnormal circadian rhythm [167]. 

Until now, some studies have suggested that long-term environmental exposure to darkness might induce hyperandrogenism via melatonin receptor 1 and reduced expression of aromatase [165]. Melatonin receptors belong to transmembrane G-protein-coupled receptors, and two subtypes in humans and other mammals can be distinguished: melatonin receptor 1 (MT1; MTNR1A) and melatonin receptor 2 (MT2; MTNR1B) [171]. In vitro experiments on the KGN cell line have demonstrated that long-term darkness leads to estrous cycle disorder, PCOM, increased LH levels as well as the LH:FSH ratio, hyperandrogenism, and glucose intolerance [165]. Furthermore, decreased expression of MTNR1A in rat ovarian GCs was also noted in darkness-treated cells [165]. The decrease in MTNR1A inhibited the androgen receptor (AR) and the expression of CYP19A1 (aromatase). The authors suggested that altered expressions of MTNR1A and AR play a crucial role in the pathological development of hyperandrogenisms [165]. These findings were in accordance with changes in hGCs collected during the oocyte retrieval process from women with PCOS, who underwent in vitro fertilization and embryo transfer [165]. On the other hand, rescue treatment with a melatonin receptor agonist and restoration of the normal light/dark circadian rhythm has partially alleviated reproductive abnormalities, such as estrous cycle disturbance and PCOM, and endocrinal hormone balance in rats treated with long-term darkness [165].

Furthermore, recent studies have revealed the association between common genetic variations of the melatonin receptor, such as single nucleotide polymorphisms (SNPs) rs2119882 as well as rs10830963, and the prevalence of PCOS [172,173]. In addition, Wang et al. have described a significant association between the rs10830963 SNP and concentrations of testosterone in women with PCOS [174].

The master pacemaker of the circadian clock in hypothalamic suprachiasmatic nucleus (SCN), modulates the circadian cycle through a rhythmic secretion of regulatory hormones such as melatonin and corticotropin-releasing hormone (CRH)/adrenocorticotropic hormone (ACTH) [175,176]. In fact, the central circadian clock regulates pineal melatonin secretion. The levels of melatonin are modulated by photoperiod; the secretion is enhanced at night in response to darkness, while bright light directly inhibits its production [177].

Nevertheless, melatonin is also produced in other tissues and organs such as the skin, gastrointestinal tract, retina, bone marrow, and lymphocytes [178,179]. Interestingly, there is emerging evidence that melatonin synthetic enzymes such as arylalkylamine N-acetyl-transferase and hydroxyindole-O-methyltransferase are present in most tissues, including ovaries and follicular cells, oocytes, and cytotrophoblasts [178,180]. 

Until now, several studies have noted an altered melatonin rhythm in women with PCOS [181,182]. It has been revealed that levels of melatonin and its metabolites, such as 6-sulphatoxymelatonin (aMT6s), are significantly elevated in the serum and urine of PCOS patients, particularly at night [183,184,185]. aMT6s is one of the major metabolites of melatonin, which can serve as an accurate marker for melatonin production [186]. On the contrary, a reduction in melatonin levels was reported in follicular fluid from women with PCOS [187,188]. Due to its antioxidant properties, melatonin is known to protect the follicles against oxidative stress and atresia; thus, melatonin plays an important role during ovulation [184]. It has been revealed that deficiency of melatonin leads to disturbance of gonadotropin secretion and alteration of the LH:FSH ratio, the remarkable features in women with PCOS [189].

Another study has revealed that increased serum concentrations of melatonin in PCOS patients were associated with testosterone levels [184]. Furthermore, it has also been highlighted that the night-time urine levels of aMT6s and 8-hydroxy-2′-deoxyguanosine (8-OHdG) were significantly elevated in women with PCOS compared to those in the control group. In contrast, the day-time urine levels of aMT6s and 8-OHdG were comparable to healthy women [185]. 8-OHdG is a product of free radical-induced oxidative damage to 2′-deoxyguanosine. It has been widely used as a marker for assessing oxidative stress and carcinogenesis, since it can be detected in urine [185]. Higher levels of aMT6s at night are suggested to be a result of increased melatonin secretion in response to increased oxidative stress in women with PCOS [190]. Furthermore, melatonin levels have also been shown to be inversely correlated with the serum LH:FSH ratio in PCOS patients [184]. There is emerging evidence that supplementation with melatonin can improve the oocyte and embryo quality in PCOS women, and could be a good strategy in the management of hormonal aberrations as well as insulin resistance associated with PCOS.

## 4. Endocrine Disrupting Chemicals (EDCs)

Among EDCs, ovarian disruptors can be distinguished. Studies have pointed out the negative effects on ovarian function of plasticizers (e.g., bisphenol A and phthalates), pesticides (e.g., dichlorodiphenyltrichloroethane and methoxychlor), dioxins, polychlorinated biphenyls, pharmaceutical agents (diethylstilbestrol), and phytoestrogens such as genistein [191]. The evidence for the relationship between numerous disorders and exposure to EDCs is further supported by several studies. These compounds, such as bisphenol A or organochlorine pesticides, have been reported to act like xenohormones in women due to estrogen-like activity and/or anti-testosterone action, but also by altering FSH and LH secretion [24]. Although previously it was suggested that EDCs might interact only via nuclear receptors, e.g., sex steroid receptors, several studies have indicated that EDCs might also act through membrane receptors, neurotransmitter receptors, orphan receptors, and pathways associated with hormone synthesis [192]. Accumulating data indicate the potential role of EDCs in several aspects of female reproductive disorders; thus, it might be assumed that these substances have also targeted the metabolic and reproductive features of PCOS.

In the following section, we have presented experimental evidence connecting EDCs with metabolic and reproductive derangements resembling the clinical manifestations of PCOS.

### 4.1. Reproductive and Neuroendocrine Dysfunctions Associated with Exposure to EDCs

**Figure 3:**
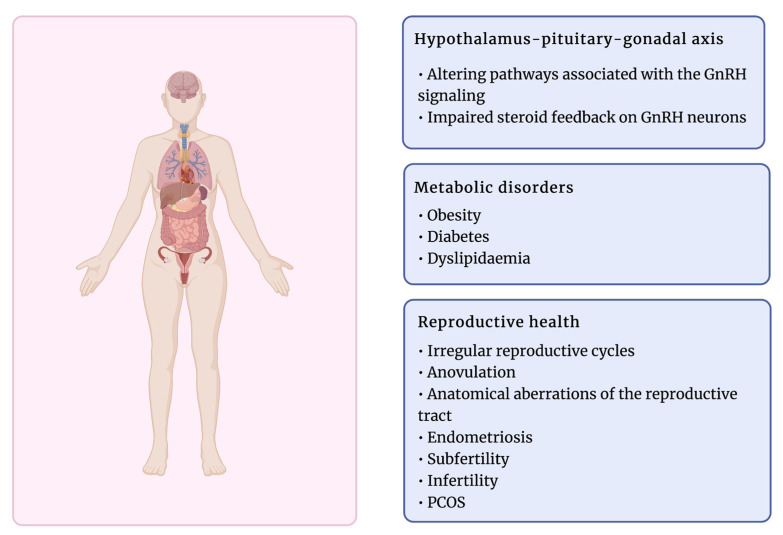
The effects of exposure to EDC on female health. Created with BioRender.com.

Exposure to EDCs has been associated with various reproductive dysfunctions (Figure 3). A growing number of studies have shown that these compounds can affect hormone receptors by agonist or antagonist activity, lead to anovulation, anatomical aberrations of the reproductive tract, or other disorders such as endometriosis and subfertility [24,193]. Therefore, the female reproductive system is exceptionally susceptible to chemical compounds, and the time of exposure to EDCs determines the effect of their activity, particularly during fetal development [79]. EDCs have been suggested to target ovarian functions directly and indirectly by targeting pivotal neuroendocrine functions at the hypothalamus–pituitary level. Thus, both follicular growth and steroid hormone secretion might be interrupted [192,194]. Several EDCs have been reported to alter the ovary response to gonadotropin stimulation due to affecting the gonadotropin receptor function, which binds pituitary hormones LH and FSH [192,195].

EDCs are known to act through numerous mechanisms, altering the pathways associated with GnRH signaling. EDCs, such as BPA, methoxychlor, or polychlorinated bisphenyls have been revealed to alter the expression of the estrogen-sensitive neuropeptide—kisspeptin, involved in the regulation of GnRH, but also via the direct influence on GnRH neuron expression in the hypothalamus and the impaired steroid feedback on GnRH neurons [195].

Overall, the complexity of the multiple mentioned mechanisms contributes to the disturbance of sensitive endocrine balance.

### 4.2. Bisphenol A

Bisphenol A (BPA) is an organic synthetic chemical that belongs to the group of diphenylmethane derivatives and bisphenols (4,40-dihydroxy-2,2-diphenylpropane) abundantly present in the environment [80]. BPA is used as a co-monomer mainly in producing polycarbonate plastic. It has many applications, including use in plastic containers, baby bottles, medical devices, or food and beverage can inner liners [81]. BPA has been suggested to have estrogenic properties, as its chemical structure (two phenyl rings connected to the methyl groups) resembles the estrogen scaffold [196]. Accumulating evidence suggests that BPA has weak estrogen activity since it can bind to both ERα and ERβ nuclear receptors, however, to a much lesser extent than 17β-estradiol [196,197]. Interestingly, BPA has been revealed to act as an estrogen agonist or antagonist, depending on molecular environments [198]. Exposure to BPA is a growing and important health concern since BPA can seep into food and beverages from plastic containers made with BPA. Moreover, it can also be inhaled or pass through the epidermis [199].

According to this assumption, significant levels of BPA have been revealed in the blood and biological fluids, including ovarian follicular fluid [81,200,201], supporting the hypothesis that BPA might affect ovarian follicles and reduce ovarian reserve [82,200].

In rats, neonatal exposure to BPA has been associated with a higher risk of development of the PCOS reproductive phenotype in adulthood [202]. In addition, BPA has been revealed to disrupt neuroendocrine and ovarian function, alter metabolism, and affect fertility in an animal model [203]. The studies suggest that developmental exposure to BPA in a dose-dependent manner might impair ovarian follicular development in rodents, resulting in a higher number of antral follicles, however, with decreased corpora lutea formation [204,205]. Furthermore, a reduced number of ovulated oocytes was noted after exposure to low doses of BPA, similar to those in the environment [205]. In another study, prepubertal exposure to BPA in rats decreased the expression of genes that promote follicle development and, conversely, increased the expression of the AMH gene, which is involved in inhibiting follicular development [206].

Furthermore, a significant cytotoxic effect on human GCs after exposure to BPA in concentration of 25 µM was found in our preliminary studies—the cell survival was decreased to 86% [unpublished data].

Studies conducted in the culture of antral follicles isolated from 32-day-old mice have reported that BPA inhibits ovarian steroidogenesis and decreases antral follicle development in vitro, as evidenced by reduced levels of progesterone, DHEA, androstenedione, estrone, testosterone, and estradiol, but also decreased expression of *STAR*, *HSD3B1*, and *CYP17A1* [207]. However, accumulating data suggest that BPA contributes to increased androgen levels directly by stimulating the ovarian theca cells, and indirectly through interactions with GCs [208]. The results reported by Zhou et al. have described dose-dependent changes in sex steroid levels and mRNA steroidogenic enzymes in theca interstitial and GC cultures treated with BPA [82]. This study has indicated increased testosterone synthesis and mRNA expression of 17α-hydroxylase (*P450c17*) and cholesterol side-chain cleavage enzyme (*P450scc*) in theca-interstitial cells, which are suggested to be key features associated with PCOS pathogenesis. Furthermore, another study has revealed that BPA, in a dose-dependent manner, enhanced basal (1mM) and FSH-induced (10 mM) progesterone synthesis in GCs, simultaneously suppressing FSH-stimulated estradiol production [209].

Déchaud et al. have also revealed that BPA is capable of binding to human sex hormone binding globulin (SHBG), which then transports BPA through the plasma. Thus, as a result, BPA might target tissues that express estrogen receptors. Moreover, the study has indicated that BPA can remove sex hormones from SHBG, and consequently increase the level of circulating free androgens [210].

Subsequent studies in humans have shown that exposure to BPA in adults could be related to various reproductive dysfunctions and metabolic diseases in women and men. Accordingly, BPA exposure was associated with reduced male sexual function as well as sperm quality and reduced ovarian response, decreased fertilization rate and embryo quality, implantation failure, miscarriage, premature delivery or endometrial disorders [211].

Several studies have proven that BPA levels in blood or urine are significantly higher in PCOS women compared to controls, and are also positively correlated with androgen levels [212,213,214,215]. These results indicate that BPA can potentially contribute to ovarian hyperandrogenism via androgen metabolism disruption or their displacement from SHBG, which suggests the potential role of BPA in PCOS pathogenesis.

Interestingly, in contrast to previous findings, some studies have proposed the hypothesis that PCOS leads to higher BPA levels. This is due to the fact that an elevated amount of circulating testosterone and androgen levels in PCOS women might decrease the BPA clearance [202,216], since high androgen levels are known to reduce the activity of uridine diphosphate-glucuronosyl transferase, involved in the degradation and clearance of BPA from the circulation [217,218].

The collected data are consistent; there is a significant positive correlation between PCOS and high BPA levels in patients. However, it remains a matter of controversy whether this interplay is caused by BPA, or is a result of PCOS per se [80].

### 4.3. Phthalates

Phthalates belong to the group of industrial chemicals, frequently used as plasticizers—functional substances that increase the flexibility and durability of plastic. They are used in the production of a wide range of everyday products, such as plastic toys, food packaging, paints, plastic bags, and cosmetics [219]. The most commonly used are di-2-ethylhexyl phthalate (DEHP) and its metabolite, mono (2-ethylhexyl) phthalate (MEHP), which have been identified as EDCs. They have been associated with the pathogenesis of several health disorders, including obesity, abnormalities in genital development, low quality of semen, precocious puberty, and gynecomastia [220,221,222,223]. To date, research on the possible role of phthalates in PCOS development has mostly been limited to in vitro and animal model studies. DEHP has been shown to alter the estrous cycle and decrease the ovulation rate due to the decrease in estrogen and progesterone levels, leading to anovulatory cycles in rats and, in turn, to PCOM [224,225,226]. Decreased estradiol levels in GCs lead to a lack of LH surge, which is essential for ovulation [225].

In the other study, MEHP has been shown to stimulate steroidogenesis, decrease progesterone production, and aromatase levels in rat GCs, leading to hyperandrogenism, a cardinal feature of PCOS [227]. Similarly, Reinsberg et al. have shown that MEHP inhibited estradiol production and altered steroidogenesis in luteinized GCs derived from women who underwent the in vitro fertilization procedure [228].

To date, a few studies in humans have been conducted; however, these findings are not consistent. Akın et al. have revealed that DEHP levels, after the BMI adjustments, are associated with insulin resistance and dyslipidemia in adolescent girls with PCOS [213]. However, this study did not observe any relationship between DEHP/MEHP and gonadotropins or sex hormones [213]. These findings may not be representative due to the limitations of the study: serum FSH, LH, progesterone, and phthalate levels were not measured at the same time of the menstrual cycle in patients. However, the authors suggest that DEPH can play a role in PCOS development due to insulin resistance at the follicle level.

A recent study by Jin et al. has revealed significantly increased levels of DEHP in the follicular fluid of women with PCOS who underwent in vitro fertilization compared to the control, associated with lower pregnancy outcomes. Furthermore, DEHP treatment caused a significantly elevated androgen level in human GCs [229]. Additionally, exposure to DEHP resulted in notably lower viability of GCs and the KGN cell line, promoted apoptosis, altered expression of apoptosis-related genes, and caused cell cycle arrest [229]. On the other hand, our studies have indicated that exposure to both DEHP and MEHP in the concentration range of 12.5–400.0 µM increased cell proliferation and did not exert any cytotoxic effect on human ovarian GCs [unpublished data].

Contrary to what has been reported by Jin et al., Vagi et al. found the anti-androgenic effects of certain phthalates in PCOS patients [230]. Furthermore, DEHP and MEHP have previously been indicated to have an antiandrogenic effect in animals [231], and MEHP, as well as diisononyl phthalate (DiNP), have been reported to decrease testosterone production in men [232].

Environmental exposure to DEHP through oral ingestion, inhalation, or skin is significant. Moreover, DEHP has been shown to cross the placenta, since its metabolites have been found in amniotic fluid [233]. Based on these findings, DEHP might be suggested to exert potential reproductive and developmental toxicity. It has been shown that DEHP exerts adverse effects on puberty, fertility, pregnancy, and the overall female reproductive tract [234].

### 4.4. 2,3,7,8-Tetrachlorodibenzo-p-dioxin

2,3,7,8-tetrachlorodibenzo-p-dioxin (TCDD) is considered the most toxic member among the dioxin group of chemicals. TCDD is a persistent environmental pollutant produced as an unwanted by-product of herbicide and pesticide manufacturing. In addition, it is also a side product in paper, fungicides, and color metal production [235]. Considering its high lipid solubility, chemical stability, and resistance to elimination processes, TCDD can quickly accumulate in human and animal tissues [235]. To date, the studies have confirmed the presence of TCDD in blood serum, breast milk, and ovarian follicular fluid [236]. Furthermore, its half-life, defined as the time it takes for a quantity to reduce to half its initial value, is estimated to be relatively long in humans (7–11 years) and even up to 100 years in the environment [235,237,238]. TCDD has been associated with various negative health disorders, for instance, in occupationally exposed humans, such as pesticide producers, or after environmental disasters, such as the Vietnam War and industrial accidents [239].

There is a constantly growing body of evidence that exposure to TCDD can target ovarian function and alter folliculogenesis [240]. Several studies have revealed that exposure to TCDD exerts an antiproliferative effect on follicles in pigs [241] and decreases the number of antral follicles in rats [242]; however, it does not affect the growth of antral follicles nor the proliferation of GCs in mice [243]. Some studies suggest that the differences in response to TCDD among various species might be related to abilities to metabolize TCDD and different expressions of the AhR, the TCDD receptor [244].

Furthermore, TCDD has been revealed to disrupt/arrest ovulation in rodents in vivo [245,246], possibly by reducing the number of S-phase GCs and decreasing the levels of cyclin-dependent kinase 2 and cyclin D2 during the pregnant mare’s serum gonadotropin (PMSG) treatment. Based on these findings, the authors suggest that the inhibitory activity of TCDD might be exerted due to the attenuation of the cell cycle via the AhR-mediated cascade [246].

TCDD has also been revealed to alter ovarian steroidogenesis. The studies have shown that exposure to TCDD decreased levels of progesterone, androstenedione, testosterone, and estradiol in isolated mouse antral follicles. Interestingly, the addition of pregnenolone re-established the normal hormone levels, which suggests that pregnenolone production might be the target point of TCDD activity [243,247]. Thus, the TCDD activity seems to be associated with the inhibition of the critical steroidogenic enzymes: 17β-HSD-1 and CYP19A1 [247]. These results are in line with those obtained in the animal in vivo studies, which also described TCDD inhibitory effect on ovarian steroidogenesis [240]. In their systematic literature review, Gaspari et al. provide an insight into the transgenerational effects of TCDD on reproductive health in rodents [239]. Several studies have confirmed the transgenerational consequences of exposure to TCDD, similar to human reproductive derangements, such as pubertal abnormalities and menstrual disorders, endometriosis, premature ovarian insufficiency, PCOS, subfertility, or adverse pregnancy outcomes [239]. Since PCOS is one of the major disorders affecting women’s reproductive health, it has been widely analyzed in mammalian models [248,249]. Exposure of Sprague Dawley rats to TCDD by intraperitoneal injection (IP) resulted in a significantly decreased number of primordial follicles, as well as the PCOM (cardinal PCOS feature), in subsequent generations [250]. Moreover, the increased number of ovarian cysts was observed in the transgenerational F3 animals to a much greater extent than the indirectly exposed F1 generation, which suggests that PCOM might be explained by epigenetic transgenerational mechanisms more than direct exposure to TCDD [250].

Indeed, studies that present ovarian abnormalities in rodents are not directly correlated with the clinical aspects of human ovarian disorders. However, they could provide valuable information on the possible impact of TCDD on human reproductive health.

### 4.5. Tributyltin

Growing evidence suggests an association between exposure to tributyltin (TBT) and reproductive and metabolic features resembling those in animal models of PCOS and PCOS patients [251]. TBT is a persistent organometallic compound with many applications in agriculture and industry, such as broad-spectrum biocides, wood preservatives, and antifungal agents in textiles [252]. However, TBT was initially developed and used as an antifouling coating on boats and ships from the 1960s to the 1990s, when it became apparent that tributyltin compounds were highly toxic to various species of aquatic organisms [253]. Due to its long environmental half-life and presence in the human food chain, there is a high risk of TBT exposure, primarily due to contaminated seafood, water, and sediments [254]. Several studies have reported significantly increased TBT levels in coastal areas [255,256]. For instance, the levels of butyltin compounds in fish muscles and livers from the Polish coast of the Baltic Sea were 715 and 1132 ng Sn/g dry weight, respectively [257]. Furthermore, significant levels of TBT (50–400 nM) were also detected in human blood [258].

Several epidemiological and animal studies have demonstrated that TBT exposure is linked to reproductive and metabolic features that resemble those found in PCOS [251].

The study on adult female rats has revealed that oral administration of TBT (100 ng/kg/day) for 15 days caused irregular estrous cycles, reduced estrogen levels, low ovary weight, pyknotic nuclei of ovarian GCs, and a greater number of atretic and cystic ovarian follicles [259]. Correspondingly, the 15-day administration of the same dose of TBT was found to decrease the LH surge, GnRH expression, and susceptibility to kisspeptin in female rats. It also altered the corpora lutea (CL) formation and estrogen negative feedback, and increased testosterone levels [260,261].

Previous investigators have revealed that TBT caused pregnancy complications and failure due to the high incidence of embryo pre-implantation and post-implantation loss during the first seven days of gestation in female rats [262,263]. Furthermore, exposure to TBT was also associated with uterine irregularities. TBT caused uterine atrophy, inflammation, and a reduction in the endometrium layer, which resulted in lowering the fertility rates in female rats [260,264].

Metabolic disorders such as obesity, hyperlipidemia, insulin resistance, and compensatory or hyperinsulinemia are common features in PCOS. Several studies have suggested the obesogenic activity of TBT [251]. Obesogens induce obesity by increasing lipid storage in existing adipocytes and promoting development of new fat cells, and altering energy balance and regulation of appetite and satiety [265,266]. TBT is known to disrupt multiple signaling pathways; however, its activity is mediated mainly through peroxisome proliferator-activated receptor γ (PPAR-γ), a key regulator of adipocyte differentiation and a transcriptional regulator and/or effector of target genes CCAAT Enhancer Binding Protein Beta (C/EBP), adipocyte-specific fatty acid-binding protein (AFABP), and fatty acid transport protein (FATP) [266,267]. PPAR-γ belongs to the PPAR subfamily of nuclear hormone receptors, which are mainly present in adipose and hepatic tissue and are involved in adipocyte formation [268]. *PPAR-γ* seems to be a pivotal gene involved in the development of obesity in humans and rodents [251,266], and it was suggested that a polymorphism in exon 6 of the *PPAR-γ* gene is associated with obesity in women with PCOS [269].

Indeed, TBT was found to promote obesity, increase insulin and androgen levels, as well as alter hypothalamic–pituitary–gonadal axis function via disruption in kisspeptin/leptin signaling in female rats [260]. Interestingly, several studies have noted that in utero exposure of pregnant animals to TBT was linked to metabolic abnormalities that resemble those in PCOS [260,266,270].

The adverse effects of TBT at different levels of the reproductive system, including altered steroid profiles, dysfunctional steroidogenesis, and derangements in GCs function, are consistent with changes observed in PCOS patients, but also in DHT-induced and JCR:LA-cp rodent models of PCOS [251]. Therefore, the absence of CL formation and polycystic ovaries were noted in mice treated with dihydrotestosterone (10 mg, s.c.) for 90 days [271].

Although knowledge about the effects of TBT in animals is extensive, there is little information about its activity in humans [251]. Rantakokko et al. have revealed that placental TBT levels were related to increased weight gain of the newborn during the first three months of life [272]. Moreover, an environmentally relevant dose of TBT (1 or 10 ng/mL) was found to stimulate theca cell cholesterol extracellular efflux through the retinoid X receptor (RXR) pathway, which in consequence, induced a compensatory upregulation of STAR and SREBF1. The latter ones are responsible for the transfer of cholesterol into the mitochondria and the novo cholesterol synthesis, respectively [273].

### 4.6. Glyphosate

Glyphosate [N-(phosphonomethyl) glycine] is an active compound of glyphosate-based herbicides (GBHs), which are the most widely used pesticides in conventional agriculture worldwide. Glyphosate targets 5-enolpyruvylshikimate-3-phosphate synthase (EPSPS), the enzyme that catalyzes the penultimate step of the shikimate pathway, responsible for the biosynthesis of aromatic compounds in plants and microorganisms [274]. Based on the toxicity data and the mechanism of its activity, glyphosate is considered the “least toxic” substrate for mammals [275]. However, there is growing evidence suggesting that glyphosate and GBHs may alter endocrine balance, resulting in reproductive dysfunction [275]. On the contrary, the Endocrine Disruptor Screening Program and the European Food Safety Authority (EFSA) did not find sufficient evidence to consider glyphosate an endocrine disrupting chemical [276,277], so this issue remains contentious.

Elevated levels of glyphosate have been detected in the environment (surface waters, groundwaters, open-reservoir tank waters, soil, dust, and air), and various food products, such as soy-based infant formula and soy sauce [275]. Therefore, concern about the effects on human health is constantly growing.

Glyphosate was revealed to exhibit estrogen-like characteristics by either direct activation or inhibition of estrogen activity, and by acting indirectly by modulating its action [278]. However, its estrogenic activity seems to be weaker in comparison to E2 [275]. Several studies have demonstrated that glyphosate and GBHs decreased aromatase expression and, consequently, its activity in human embryonic kidney 293 (HEK-293) cells and human placental JEG3 cells [279,280]. Furthermore, it has been revealed that glyphosate and GBHs decrease E2 secretin from bovine and swine GCs [281,282,283]. Their estrogenic activity was also assessed in human breast cancer cells. Glyphosate was found to induce the proliferation of hormone-dependent human breast cancer cell lines T47D and MCF-7. However, it did not enhance the proliferation of the hormone-independent human breast cancer cell line MDA-MB231 [284,285]. Moreover, this compound was revealed to enhance estrogen response element (ERE)-mediated transcription of a *luciferase reporter* gene by a ligand-independent mechanism. The antiestrogen Fulvestran, in turn, was able to mitigate the proliferative and stimulatory effects of glyphosate [284,285]. Based on these results, estrogen receptor (ER) signaling might be suggested to be involved in the proliferative activity of glyphosate. Therefore, several in vivo studies have demonstrated the estrogenic properties of glyphosate. The neonatal exposure to GBH (2 mg glyphosate/kg body weight/day) was found to alter the uterus response to E2 in the later lives of rats [286]. Subsequently, studies in animal models have shown that exposure to glyphosate or GBHs altered E2 levels, ERα protein and gene expression, as well as E2-dependent gene expression [28,29,30,31,32,33,34,35,36,37,38,39,40,41,42,43,44,45,46,47,48,49,50,51,52,53,54,55,56,57,58,59,60,61,62,63,64,65,66,67,68,69,70,71,72,73,74,75,76,77,78,79,80,81,82,83,84,85,86,87,88,89,90,91,92,93,94,95,96,97,98,99,100,101,102,103,104,105,106,107,108,109,110,111,112,113,114,115,116,117,118,119,120,121,122,123,124,125,126,127,128,129,130,131,132,133,134,135,136,137,138,139,140,141,142,143,144,145,146,147,148,149,150,151,152,153,154,155,156,157,158,159,160,161,162,163,164,165,166,167,168,169,170,171,172,173,174,175,176,177,178,179,180,181,182,183,184,185,186,187,188,189,190,191,192,193,194,195,196,197,198,199,200,201,202,203,204,205,206,207,208,209,210,211,212,213,214,215,216,217,218,219,220,221,222,223,224,225,226,227,228,229,230,231,232,233,234,235,236,237,238,239,240,241,242,243,244,245,246,247,248,249,250,251,252,253,254,255,256,257,258,259,260,261,262,263,264,265,266,267,268,269,270,271,272,273,274,275,276,277,278,279,280,281,282,283,284,285,286,287,288,289,290,291]. There is little information on the effects of glyphosate exposure on ovaries, as well as granulosa and theca cell function. However, Ren et al. have revealed that mice exposed in utero to glyphosate presented a decreased ovarian weight and increased atretic follicles, together with altered estrogen and progesterone levels [289]. The authors have also observed changes in the expression profiles of several genes at the hypothalamic–pituitary–ovarian axis, such as: *GnRH*, *LHR*, *FSHR*, *3β-HSD*, and *CYP19A1* [289]. Similarly, another study has shown impaired folliculogenesis, decreased estrogen secretion, and abnormal ovarian morphology [292]. Furthermore, the study conducted in lambs exposed to GBH from birth to postnatal day 15 has revealed an increase in the number of atretic follicles, decreased mRNA levels of *FSHR* and *growth/differentiation factor 9*, and induced growth arrest in developing follicles [293].

All these features suggest that there might be a link between the endocrine disrupting activities of glyphosate as well as GBHs, and the adverse effects on female reproductive health. In addition, some of these characteristics resemble those in PCOS. Interestingly, a new possible role of glyphosate in the pathogenesis of PCOS has been discussed [294]. Recently, several studies have associated the gut microbiota disturbance with the observed clinical and pathophysiological features of PCOS [86]. Parker has suggested that glyphosate may induce intestinal permeability as a result of disturbance of the gut microbiota, which might contribute to the pathogenesis of PCOS [294].

Furthermore, glyphosate has been revealed to induce multigenerational health effects, transmitted to future generations [295,296]. Several in vitro and ex vivo studies have demonstrated the ability of glyphosate to cross the human placenta [297,298]. Therefore, glyphosate was detected in considerable concentrations in the serum of pregnant women at childbirth (0.2–189.1 µg/L), as well as in umbilical cord samples (0.2–94.9 µg/L) [299]. Epidemiological data have shown that preconception exposure to glyphosate was correlated with an increased risk of late abortions in a rural population in Canada [300]. Furthermore, elevated glyphosate urine levels were associated with a decreased gestation period in an Indiana (USA) cohort from rural as well as non-rural areas [301]. Similarly, animal studies have shown harmful effects of exposure to glyphosate and GBH on reproductive health, including pre- and post-implantation embryo loss, delayed fetal growth, and structural congenital abnormalities [275].

### 4.7. Other EDCs That Affect the Female Reproductive System

The female reproductive system is an important target for EDCs. In our review, we have presented some of the most studied EDCs (BPA, phthalates, 2,3,7,8-tetrachlorodibenzo-p-dioxin, tributyltin, and glyphosate), which are directly and/or indirectly associated with the pathogenesis of PCOS. The EDCs summarized in this paragraph are those that have been linked to reproductive or developmental disorders; however, there is little or no evidence on their connection with PCOS characteristics.

Triclocarban (TCC) is a broad-spectrum antimicrobial compound widely used in personal care products, such as dermal cleaning products, body lotions, deodorants, and wipes [302]. TCC is considered as an EDC; however, the mechanisms of its activity, especially estrogenic, are still unclear [303]. Due to concerns about human health after exposure to TCC, human studies have been performed. Geer et al. have revealed that the concentration of TCC in cord blood plasma was correlated with a decrease in gestational age at birth after prenatal exposure. Furthermore, the TCC metabolite (3′-Cl-TC), assessed in third-trimester maternal urine, was associated with fewer low birth weights [304]. On the other hand, the study by Wei et al. did not show an association between TCC levels in maternal serum or cord blood and fetal anomalies, in a cohort population from Beijing, China [305].

TCC is often discussed and studied together with triclosan (TCS), 5-chloro-2-(2,4-dichlorophenoxy)phenol; however, the biological activities of these two compounds are not the same [302]. TCS is a lipid-soluble antimicrobial compound, commonly used in various personal care, household, veterinary, and pharmaceutical products [306]. Due to its widespread use, people are exposed to TCS through dermal mucosal absorption and ingestion [307,308]. Therefore, TCS has been detected in human urine, plasma, breast milk, brain, liver, and adipose tissue [309,310,311,312,313,314]. Furthermore, several studies have noted higher concentrations of TCS in females than males [315]. Due to the structural similarities between TCS and estrogens, it can affect hormone balance through interactions with hormone receptors [315]. Thus, TCS is suggested to alter female reproductive health. Recently, several studies have revealed that TCS disturbs thyroid homeostasis, the gut microbiome, and promotes carcinogenesis in the breast, ovaries, and prostate [316,317,318]. TCS was also shown to affect luteal cell progesterone production and disrupt ovarian function [315]. According to data from the National Health and Nutrition Examination Survey, there is an association between TCS exposure and the inability to conceive over a period of one year [319]. The cross-sectional study, in which 674 infertile women were recruited, has revealed that women with PCOS had significantly higher levels of urinary TCS compared to the group without PCOS [320]. Interestingly, the authors have observed increased levels of LH and the LH/FSH ratio in healthy women, as a result of environmental exposure to TCS [320]. Furthermore, a prospective cohort study, which included 698 women, found the association between high urine triclosan levels and increased risks of abnormal menstruation, as well as a prolonged menstrual cycle. Furthermore, TCS in concentration greater than 4.5 ng/mL was correlated with a 23% reduction in fecundability compared to the lowest level of TCS [321]. On the contrary, the study by Gu et al. did not show any association between TCS in urine samples collected from 40 women with PCOS, and PCOS either in an unadjusted binary logistic regression model, or in a model adjusted for potential confounders [322].

Selected studies that have assessed the developmental and endocrine/reproductive effects of TCC and TCS in animal models are summarized in Table 1.

### 4.8. Endocrine Disrupting Chemicals and Pregnancy

It is well known that pregnancy is a period of increased susceptibility to toxicant exposure. Pregnant women are ubiquitously exposed to EDCs. Due to their ability to cross the placenta barrier, EDCs are environmental factors that could significantly affect fetal development and induce long-term consequences for infant and child health [333]. Several studies have revealed that prenatal exposure to EDCs might be related to asthma and allergies, low birth weight, prematurity, pubertal development abnormalities, neurobehavioral disorders, or breast cancer [333,334,335]. One of the recently conducted studies has revealed that more than 50% of the interviewed pregnant women had never heard of EDCs before [333].

In this context, the education of pregnant women about EDCs should be essential; thus, the health benefits gained from a reduction in pregnant women’s exposure to endocrine disruptors seem to be particularly significant.

Recently, the Royal College of Obstetricians and Gynecologists, Endocrine Society, and the International Federation of Gynecology and Obstetrics (FIGO) have recommended that all pregnant women should be informed of the possible risks of EDCs. Moreover, education programs should be developed to inform health professionals [336,337,338].

## 5. Conclusions

During the last decades, numerous scientific groups have made outstanding efforts to understand the pathogenesis of PCOS. Multiple, not mutually exclusive, mechanisms have been proposed, including the contribution of environmental factors, especially EDCs, such as bisphenol A, phthalates, dioxin, tributyltin, and glyphosate. In this article, we have provided an overview of the literature connecting exposure to selected EDCs with neuroendocrine and reproductive disorders resembling PCOS. Moreover, we have presented aberrations in theca and granulosa cell function in the development of PCOS, and discussed the possible role of EDCs in this process.

## Figures and Tables

**Table 1:** The selected developmental and endocrine/reproductive effects of TCC and TCS in animal models.

EDC	Model	Strain	Exposure Duration	Age at Exposure	Route of Exposure	Dosage	End Points	Source
TCC	Rat	Sprague Dawley	35d	Embryonic, adult	Oral (food)	0.2% *w*/*w*, 0.5% *w*/*w*	↓ body weight and survival in pups	[323]
	Rat	Wistar	21d	Embryonic, adult	Gavage	0.3 mg/kg, 1.5 mg/kg, 3 mg/kg	↓ estradiol levels in the TCC 0.3 and TCC 3.0 groups of female pups ↓ progesterone levels ↑preimplantation loss in the TCC 3.0 in adulthood	[324]
	Mouse	hUGT1*28 and CAR-null	2d	Adult	Intra-peritoneal	16 mg/kg, 20 mg/kg	↑ hUGT and CYP gene expression via the CAR	[325]
	Mouse	CD-1	GD1-18; PND0-10	Embryonic, neonate, adult	Oral (water)	100 nM	↑ body weight of pups ↓ uterine weight in female offspring ↓ leptin, adiponectin and PPARα gene expression in adipose and liver tissues	[326]
	Fish	Zebrafish (*Danio rerio*)	24 h	Embryonic	Submersion	0.25 µM	↑ E2-induced AroB expression ↓ BPA-induced AroB expression	[327]
	Fish	Fathead minnow (*Pimephales promelas*)	22d	Adult	Submersion	1.5 µg/L	No changes in adult body weight.	[328]
	Rat	Sprague Dawley	GD5-PND21	Embryonic, lactational	Oral (water)	0.5 mg/L	↓follicle count, proliferation and gonadosomatic index of GCs. Delayed puberty onset. ↓ transition of the primordial follicles to more developed ↑ atresia, apoptosis, AR expression in GCs	[329]
TCS	Fish	Zebrafish (*Danio rerio*)	42d	Adult	TCS solution in exposure tank	0, 17, 34, or 68 µg/L	↓ expression of SOD, GPx1a, CAT, sMT-B in the ovary of 68 µg/L group ↑ oxidative damage in ovaries ↑ ROS-dependent ovary apoptosis	[330]
	Rat	Holtzman	GD6-PD21	Gestational, lactational	subcutaneous injection	0.1, 4, 40 and 150 mg/kg b. wt./day	↓ reproductive functions and fertility of F1 male rats ↓ testosterone, sperm count and motility ↓ AR, ERα and ERβ, SAR, aromatase expression ↑ pre- and post-implantation loss	[331]
	Mouse	ICR mice	50d	Adult	Oral	1, 10 or 100 mg/kg/day	↓ LH, FSH, progesterone serum levels ↓ GnRH mRNA expression ↑ PRL ↓ kisspeptin immunoreactivity	[332]

Abbreviations: “↓” stands for “decreased”; “↑” stands for “increased”; TCC—Triclocarban; TCS—Triclosan; GD—gestation day; PND—postnatal day; PPARα—Peroxisome proliferator-activated receptor α; E2—estradiol; AroB—CYP19a1; BPA—bisphenol A; GCs—granulosa cells; AR—androgen receptor; SOD—superoxide dismutase; GPx1a—glutathione peroxidase 1a; CAT—catalase; ROS—reactive oxygen species; ERα—estrogen receptor α; ERβ—estrogen receptor β; LH—luteinizing hormone; FSH—follicle stimulating hormone; GnRH—gonadotropin-releasing hormone; PRL—prolactin; hUGT: humanized uridine 5′-diphosphoglucuronosyltransferase; CAR: constitutive active/androstane receptor.

## Data Availability

Not applicable.
